# Comparison of nifekalant and amiodarone for resuscitation of out-of-hospital cardiopulmonary arrest resulting from shock-resistant ventricular fibrillation

**DOI:** 10.1007/s00540-013-1775-5

**Published:** 2014-01-05

**Authors:** Nobuya Harayama, Shun-ichi Nihei, Keiji Nagata, Yasuki Isa, Kei Goto, Keiji Aibara, Masayuki Kamochi, Takeyoshi Sata

**Affiliations:** 1 Critical Care Medicine, University Hospital, University of Occupational and Environmental Health, 1-1 Iseigaoka, Yahatanishi-ku, Kitakyushu, Fukuoka 807-8555 Japan; 2 Department of Anesthesiology, School of Medicine, University of Occupational and Environmental Health, Kitakyushu, Japan

**Keywords:** Nifekalant, Amiodarone, Out-of-hospital cardiopulmonary arrest, Ventricular fibrillation, Cardiopulmonary resuscitation

## Abstract

**Purpose:**

Nifekalant is a pure potassium channel blocker that has been used to treat ventricular tachyarrhythmias since 1999 in Japan. Intravenous amiodarone was approved later than nifekalant in Japan, and it is still unclear which of the two agents is superior. The aim of this study was to compare the efficacy of nifekalant and amiodarone for resuscitation of out-of-hospital cardiopulmonary arrest caused by shock-resistant ventricular fibrillation.

**Methods:**

From December 2005 to January 2011, ambulance services transported 283 out-of-hospital cardiopulmonary arrest patients to our hospital. Of these, 25 patients were treated with nifekalant or amiodarone in response to ventricular fibrillation that was resistant to two or more shocks. We undertook a retrospective analysis of these 25 patients.

**Results:**

We enrolled 20 men and 5 women with a mean age (± standard deviation) of 61.1 ± 16.4 years. All 25 patients were treated with tracheal intubation and intravenous epinephrine. Fourteen patients received nifekalant and 11 patients received amiodarone. The rates of return of spontaneous circulation (ROSC) (nifekalant, 5/14, versus amiodarone, 4/11; *P* = 0.97) and survival to discharge (nifekalant, 4/14, versus amiodarone, 2/11; *P* = 0.89) were not significantly different between the two groups. The time from nifekalant or amiodarone administration to ROSC was 6.0 ± 6.6 and 20.3 ± 10.0 min, respectively, which was significantly different (*P* < 0.05).

**Conclusion:**

In this small sample size study, nifekalant, compared with amiodarone, is equally effective for ROSC and survival to discharge after shock-resistant ventricular fibrillation and can achieve ROSC more quickly. Further prospective studies are needed to confirm our results.

## Introduction

Ventricular fibrillation is one of the most common life-threatening tachyarrhythmias, especially among out-of-hospital cardiopulmonary arrest patients. Because of the increased availability of automated external defibrillators in public locations, the number of out-of-hospital cardiopulmonary arrest patients with ventricular fibrillation receiving pre-hospital direct current (DC) shocks is increasing, and the survival rate of these patients has improved [1–3]. Patients with shock-resistant ventricular fibrillation need additional antiarrhythmic drug therapy, but the best protocol for this has not yet been established.

The ARREST study showed that, in patients experiencing out-of-hospital cardiopulmonary arrest because of shock-resistant ventricular fibrillation, treatment with amiodarone resulted in a higher rate of survival to hospital admission compared with placebo [4]. Amiodarone is currently widely used for the treatment of shock-resistant ventricular fibrillation [5, 6].

In Japan, both amiodarone and nifekalant are used as intravenous class III antiarrhythmic drugs. Nifekalant is a pure potassium channel blocker with a pyrimidinedione structure that was developed in Japan and has been used for the treatment of life-threatening ventricular tachyarrhythmia since 1999 [7]. Because intravenous amiodarone was approved later than nifekalant in Japan, nifekalant has been widely used as a class III antiarrhythmic intravenous drug. Amiodarone has various effects on ion channels, receptors, sympathetic activity, and thyroid function [8, 9], but nifekalant is a pure potassium channel blocker, specifically blocking the rapid component of delayed rectifier potassium currents (*I*_Kr_) without blocking sodium or calcium channels [10]. In terms of pharmacological properties, nifekalant seem to have some advantages for use in cardiopulmonary resuscitation compared with amiodarone as it does not have a negative inotropic effect [11, 12].

The ALIVE study showed that amiodarone treatment improved hospital survival rate compared with lidocaine treatment in patients with out-of-hospital cardiopulmonary arrest caused by shock-resistant ventricular fibrillation [13]. It has been reported that not only amiodarone but also nifekalant is superior to lidocaine for resuscitation of shock-resistant ventricular fibrillation [14–16], but it is still unclear which is superior, nifekalant or amiodarone. To clarify this issue, we performed a retrospective study to compare the efficacy of nifekalant versus amiodarone for the resuscitation of out-of-hospital cardiopulmonary arrest caused by shock-resistant ventricular fibrillation.

## Patients, materials, and methods

We performed a retrospective review of 283 consecutive out-of-hospital cardiopulmonary arrest patients transported to our hospital by ambulance from December 2005 to January 2011. Cardiopulmonary resuscitation (CPR) was performed according to the 2005 International Consensus on Cardiopulmonary Resuscitation and Emergency Cardiovascular Care Science with Treatment Recommendations (CoSTR) by the International Liaison Committee on Resuscitation (ILCOR), with a protocol of one shock followed by 2 min of chest compression [17]. All physicians who treated the patients were staff doctors working in critical care medicine. Of the 283 patients, 50 had ventricular fibrillation in the emergency room, including 25 with shock-resistant ventricular fibrillation (defined as ventricular fibrillation resistant to two or more shocks in the emergency room). The 25 patients with shock-resistant ventricular fibrillation were enrolled in this study. All 25 patients were treated with tracheal intubation and intravenous epinephrine (1 mg every 3–5 min) before antiarrhythmic drug administration. The physicians treating the patient decided which class III antiarrhythmic drug to use (nifekalant or amiodarone) and the dosage. The class III antiarrhythmic drug was administrated by slow intravenous injection within 1 min. The treating physicians also made decisions regarding additional treatments, including the use of extracorporeal life support (ECLS) for the patients who could not obtain return of spontaneous circulation (ROSC) after class III antiarrhythmic drug administration. We used ECLS according to the decision of physicians at the scene of CPR, based on the patient’s age, cause of cardiopulmonary arrest, presence of collapse witness, and bystander CPR. Survivors with ROSC underwent hypothermia therapy in the intensive care unit (ICU).

We compared the nifekalant and amiodarone groups in terms of (1) age, (2) gender, (3) causes of cardiopulmonary arrest, (4) rate of witnessed collapse, (5) rate of bystander CPR, (6) mean time from emergency call to paramedic arrival at the patient’s side, (7) mean time from paramedic arrival at the patient’s side to hospital arrival, (8) number of DC shocks before antiarrhythmic drug use, (9) total epinephrine dose, and (10) mean time from hospital arrival to class III antiarrhythmic drug administration.

To evaluate the efficacy of nifekalant versus amiodarone, we also compared the groups in terms of rate of ROSC, rate of survival to hospital discharge, mean time from the initiation of the drug administration to ROSC, number of DC shocks after antiarrhythmic drug use, and neurological outcome at hospital discharge as estimated using the Glasgow Outcome Scale (GOS) [18]. Statistical analyses were done with StatMate III for Macintosh (ATMS, Tokyo, Japan).

All parameters are described as mean ± standard deviation (SD). Statistical analyses were performed using the chi-square test and Student’s unpaired *t* test. A *P* value of <0.05 was considered statistically significant.

This study was carried out in accord with the principles of the Declaration of Helsinki and was approved by our University Ethics committee. The ethics committee does not require informed consent for retrospective studies such as this study.

## Results

A total of 25 patients with shock-resistant ventricular fibrillation were enrolled in this study. Of these patients, 20 were male and 5 were female, with a mean age (±SD) of 61.1 ± 16.4 years. The initial class III antiarrhythmic drug administered was nifekalant in 14 patients and amiodarone in 11 patients. There were no significant differences in the clinical characteristics of patients in the nifekalant and amiodarone groups (Table 1).

**Table 1:** Clinical characteristics of patients with out-of-hospital cardiopulmonary arrest resulting from shock-resistant ventricular fibrillation treated with nifekalant or amiodarone

	Nifekalant group (*n* = 14)	Amiodarone group (*n* = 11)	*P* value
Male	78.6 % (11/14)	81.9 % (9/11)	0.84
Age (years)	57.2 ± 16.8	66.0 ± 15.3	0.19
Causes of CPA			
IHD	71.5 % (10/14)	72.7 % (8/11)	0.71
Cardiomyopathy	21.4 % (3/14)	9.1 % (1/11)	0.40
Trauma	7.1 % (1/14)	18.2 % (2/11)	0.64
Presence of collapse witness	50.0 % (7/14)	63.6 % (7/11)	0.50
Presence of bystander CPR	57.1 % (8/14)	45.5 % (5/11)	0.86
Time interval (min)			
Emergency call–arrival of paramedics at the scene of CPA	8.3 ± 3.0	8.0 ± 4.0	0.84
Arrival of paramedics at the scene of CPA–hospital arrival	15.1 ± 4.4	14.2 ± 3.3	0.55
Hospital arrival–antiarrhythmic drug use	14.5 ± 7.2	16.1 ± 7.1	0.59
Number of DC shocks before antiarrhythmic drug use (times)	3.1 ± 1.2	3.1 ± 0.9	0.91
Dose of epinephrine before antiarrhythmic drug use (mg)	2.6 ± 1.4	2.9 ± 1.9	0.69

Values are given as mean ± SD

*CPA* cardiopulmonary arrest, *DC* direct current, *ICU* intensive care unit, *IHD* ischemic heart disease, *SD* standard deviation

The initial dose of nifekalant was 12.7 ± 6.1 mg and that of amiodarone was 179.5 ± 68.8 mg. Table 2 shows the therapeutic results of the antiarrhythmic drugs. Among the 14 patients in the nifekalant group, 5 achieved ROSC, 5 had continued ventricular fibrillation, and 4 had pulseless electrical activity (PEA) after the initial dose. Among the 11 patients in the amiodarone group, 4 achieved ROSC, 2 patients had continued ventricular fibrillation, 3 patients had PEA, and 2 patients had asystole after the initial dose. The rate of ROSC was 35.7 % (5/14) in the nifekalant group and 36.3 % (4/11) in the amiodarone group. The rate of survival to discharge was 28.6 % (4/14) in the nifekalant group and 18.2 % (2/11) in the amiodarone group. These differences were not significant. Two patients in the nifekalant group and no patients in the amiodarone group survived without brain damage (GOS 5).

**Table 2:** Therapeutic results of patients with out-of-hospital cardiopulmonary arrest resulting from shock-resistant ventricular fibrillation treated with nifekalant or amiodarone

	Nifekalant group (*n* = 14)	Amiodarone group (*n* = 11)	*P* value
Dose of antiarrhythmic drug (mg)			
Nifekalant	12.7 ± 6.1		
Amiodarone		179.5 ± 68.8	
Therapeutic results of antiarrhythmic drug (%)			
ROSC and admission to ICU	35.7 % (5/14)	36.3 % (4/11)	0.97
VF	35.7 % (5/14)	18.2 % (2/11)	0.33
PEA	28.6 % (4/14)	27.3 % (3/11)	0.94
Asystole	0.0 % (0/14)	18.2 % (2/11)	0.10
Number of DC shocks after antiarrhythmic drug use (times) (excluded patients who continued VF)	1.6 ± 1.1 (*n* = 9)	1.8 ± 1.4 (*n* = 9)	0.71
Time interval (min)			
Antiarrhythmic drug use–ROSC	6.0 ± 6.6 (*n* = 5)	20.3 ± 10.0 (*n* = 4)	0.04*
Survival to discharge (%)	28.6 % (4/14)	18.2 % (2/11)	0.89
Discharge with no brain damage (GOS 5)	14.3 % (2/14)	0.0 % (0/11)	0.19
Discharge with vegetative state (GOS 2)	14.3 % (2/14)	18.2 % (2/11)	0.79

Values are given as mean ± SD

*DC* direct current, *ECLS* extracorporeal life support, *GOS* Glasgow outcome scale, *ICU* intensive care unit, *PEA* pulseless electrical activity, *ROSC* return of spontaneous circulation, *SD* standard deviation, *VF* ventricular fibrillation

* Statistically significant at *P* < 0.05

The time from the initiation of class III antiarrhythmic drug administration to ROSC was 6.0 ± 6.6 min for nifekalant (*n* = 5) and 20.3 ± 10.0 min for amiodarone (*n* = 4); this was a significant difference (*P* < 0.05).

The number of shocks administered after drug administration until the termination of ventricular fibrillation was 1.6 ± 1.1 in the nifekalant group (*n* = 9) and 1.8 ± 1.4 in the amiodarone group (*n* = 9); this difference was not significant.

Four patients who had continued ventricular fibrillation after class III antiarrhythmic drug administration were treated with ECLS. The mean time from hospital arrival to the start of ECLS was 62.3 ± 43.3 min.

## Discussion

The present study evaluates the different clinical properties of nifekalant and amiodarone for resuscitation of shock-resistant ventricular fibrillation. We compared the efficacy of nifekalant and amiodarone for out-of-hospital shock-resistant ventricular fibrillation. There were no significant differences in the rates of ROSC or survival to discharge between the two drugs, but nifekalant achieved faster ROSC than amiodarone. Because early ROSC is one of the most important factors for minimizing brain damage in cardiopulmonary arrest patients [19, 20], nifekalant has the potential to be superior to amiodarone for resuscitation of shock-resistant ventricular fibrillation. In this study, two patients in the nifekalant group and no cases in the amiodarone group returned to normal life without brain damage (GOS 5).

To the best of our knowledge, there are only two previous studies directly comparing the efficacy of nifekalant versus amiodarone for the treatment of ventricular fibrillation: one is a human study reported by Amino et al. [21] and the other is an animal model of cardiac arrest reported by Ji et al. [22].

The study by Amino et al. [21] did not show significant differences in the success rate of defibrillation or the rate of survival to discharge between nifekalant and amiodarone in patients with out-of-hospital cardiopulmonary arrest caused by to shock-resistant ventricular fibrillation. They did, however, find that it took longer from amiodarone administration to defibrillation success (33 ± 22.8 min) than from nifekalant administration to defibrillation success (10 ± 10.0 min). This finding is consistent with the results of the present study, which found that nifekalant achieved faster ROSC after shock-resistant ventricular fibrillation compared with amiodarone. Our results show a slightly shorter interval from drug administration to ROSC for both nifekalant (6.0 ± 6.6 min) and amiodarone (20.3 ± 10.0 min) compared with the study by Amino et al., possibly because of differing CPR protocols. We performed CPR according to the 2005 CoSTR by ILCOR, with a protocol of one shock followed by 2 min of chest compressions [17], and Amino et al. performed CPR according to their original method, with a protocol of one shock followed by 5 min of chest compressions. The 2010 CoSTR [23] also has a protocol of one shock followed by 2 min of chest compressions. We think that the shock should be delivered quickly after antiarrhythmic drug administration to achieve ROSC, even in patients who initially have shock-resistant ventricular fibrillation.

Ji et al. [22] reported on the efficacy of nifekalant and amiodarone in a porcine model of cardiac arrest from ventricular fibrillation. The rates of ROSC and 24 h survival were comparable between nifekalant and amiodarone. Their results indicated that the efficacy of nifekalant for resuscitation resulting from ventricular fibrillation was not inferior to amiodarone. Interestingly, the coronary perfusion pressure was significantly lower in the amiodarone group than in the nifekalant group at 30 min after successful resuscitation. Although it is difficult to evaluate hemodynamic parameters such as coronary perfusion pressure during and immediately after CPR in humans, differences in coronary perfusion pressure with the use of different antiarrhythmic drugs may influence the recovery time from cardiac arrest to ROSC.

Although amiodarone is used for treating fatal ventricular tachyarrhythmias, it is known that it occasionally causes hypotension and bradycardia [4, 24]. Amiodarone has vasodilatory and negative inotropic qualities resulting from its sodium and calcium channel-blocking effects. Amiodarone also blocks α- and β-receptors. Vasodilation and negative inotropic activity are thought to be undesirable for resuscitation. On the other hand, nifekalant is a pure potassium channel blocker, with no negative inotropic activity and almost no influence on hemodynamic state [12]. Because vasodilation and negative inotropic activity are thought to be undesirable for resuscitation, nifekalant seems to have some advantage for resuscitation from a pharmacological aspect.

Different pharmacodynamics between nifekalant and amiodarone could affect the time to achieve ROSC. Nifekalant has a rapid action and clearance with a short half-life; the elimination half-life of nifekalant is 1.53–2.07 h in healthy subjects [25, 26]. On the other hand, a long serum half-life (>14 days) was observed for amiodarone [27]. Because the effect of amiodarone continues much longer than that of nifekalant, amiodarone is still blocking sodium and calcium channels strongly even if ventricular fibrillation is terminated after a DC shock.

The defibrillation threshold is an important factor in the success of resuscitation when treating shock-resistant ventricular fibrillation. Nifekalant decreases the defibrillation threshold of ventricular fibrillation [28] but amiodarone does not [29, 30]. Although nifekalant and amiodarone have different effects on the defibrillation threshold, our clinical data showed that the defibrillation success rate (the rate of ROSC) was the same for both. There was also no difference between the groups in the number of shocks from the time of drug administration to the termination of ventricular fibrillation (nifekalant, 1.6 ± 1.1, versus amiodarone, 1.8 ± 1.4), but nifekalant did achieve faster ROSC than did amiodarone. As a shock was delivered every 2 min if needed according to the 2005 CoSTR protocol, these results indicate that both nifekalant and amiodarone terminate ventricular fibrillation on average after one or two shocks following drug administration, but amiodarone causes a longer period of asystole or PEA before ROSC than nifekalant.

The main reason for the difference between nifekalant and amiodarone in the time from drug administration to ROSC is the different effects they have, theoretically, on sodium and calcium channels. Nifekalant is a pure potassium channel blocker and has no effect on sodium or calcium channels, but amiodarone is a multichannel blocker, including potassium, sodium, and calcium channel-blocking effects. Amiodarone strongly suppresses the sinus node pacemaker function by blocking sodium and calcium channels. Even if ventricular fibrillation is terminated after a DC shock, it might be easy to induce asystole or PEA after amiodarone because the sinus node is suppressed. Negative inotropic activity by amiodarone prolongs the time to ROSC. Amiodarone also decreases systemic vascular resistance by blocking calcium channels. Low left ventricular output and dilatation of resistance vessels causes low coronary perfusion pressure. Because of the long serum half-life of amiodarone, the effect of amiodarone continues for a long time.

There are some limitations to this study. This is a single-center retrospective study with a small number of patients, and the doses of nifekalant and amiodarone varied between patients. A large prospective study is needed to determine whether nifekalant or amiodarone is superior for resuscitation of shock-resistant ventricular fibrillation. The SOS-KANTO study group is planning to start a comparison of nifekalant versus amiodarone in patients with out-of-hospital shock-resistant ventricular fibrillation by the CPR method according to the 2010 CoSTR [31].

In conclusion, in this small sample size study, nifekalant, compared with amiodarone, is equally effective for ROSC and survival to discharge after shock-resistant ventricular fibrillation and can achieve ROSC more quickly. Further clinical investigations are necessary to evaluate the effect of nifekalant compared with amiodarone.
